# Expansion, mosaicism and interruption: mechanisms of the CAG repeat mutation in spinocerebellar ataxia type 1

**DOI:** 10.1186/s40673-016-0058-y

**Published:** 2016-11-22

**Authors:** Cara Kraus-Perrotta, Sarita Lagalwar

**Affiliations:** 1 Department of Biology, Skidmore College, 815 North Broadway, Saratoga Springs, NY 12866 USA; 2 Neuroscience Program, Skidmore College, 815 North Broadway, Saratoga Springs, NY 12866 USA

## Abstract

Spinocerebellar ataxia type 1 (SCA1) is an autosomal dominant neurodegenerative disorder that primarily affects the cerebellum and brainstem. The genetic mutation is an expansion of CAG trinucleotide repeats within the coding region of the ataxin-1 gene, characterizing SCA1 as a polyglutamine expansion disease like Huntington’s. As with most polyglutamine expansion diseases, SCA1 follows the rules of genetic anticipation: the larger the expansion, the earlier and more rapid the symptoms. Unlike the majority of polyglutamine expansion diseases, the presence of histidine interruptions within the polyglutamine tract of ataxin-1 protein can prevent or mitigate disease.

The present review aims to synthesize three decades of research on the ataxin-1 polyglutamine expansion mutation that causes SCA1. Data from genetic population studies and case studies is gathered along with data from manipulation studies in animal models. Specifically, we examine the molecular mechanisms that cause tract expansions and contractions, the molecular pathways that confer instability of tract length in gametic and somatic cells resulting in gametic and somatic mosaicism, the influence of maternal or paternal factors in inheritance of the expanded allele, and the effects of CAT/histidine interruptions to the ataxin-1 allele and protein product.

Our review of existing data supports the following conclusions. First, polyCAG expansion of gametic alleles occur due to the failure of gap repair mechanisms for single or double strand breaks during the transition from an immature haploid spermatid to a mature haploid sperm cell. Equivalent failures were not detected in female gametic cells. Second, polyCAG expansion of somatic alleles occur due to hairpins formed on Okazaki fragments and slipped strand structures due to failures in mismatch repair and transcription-coupled nucleotide excision repair mechanisms. Third, CAT trinucleotide interruptions, which code for histidines in the translated protein, attenuate the formation of slipped strand structures which may protect the allele from the occurrence of large expansions. Many of the mechanisms of expansion identified in this review differ from those noted in Huntington’s disease indicating that gene -or sequence-specific factors may affect the behavior of the polyCAG/glutamine tract. Therefore, synthesis and review of research from the SCA1 field is valuable for future clinical and diagnostic work in the treatment and prevention of SCA1.

## Background

Trinucleotide repeat disorders include a number of progressive neurodegenerative disorders caused by the expansion of microsatellite repeat regions in coding and non-coding regions of specific genes. Polyglutamine diseases represent a subset of these disorders in which the repetitive sequence presents as CAG repeats. There are presently nine known polyglutamine disorders: Huntington’s disease, spinal and bulbar muscular atrophy, dentatorubral-pallidoluysian atrophy, and the spinocerebellar ataxias type 1, 2, 3, 6, 7, and 17 [1].

First characterized by John Schut in 1950, spinocerebellar ataxia type 1 (SCA1) is an autosomal dominant neurodegenerative disease characterized by progressive ataxia and eventual deficits with eye movement, speech, and swallowing [2]. Symptoms are accompanied by degeneration in cerebellar Purkinje neurons and brainstem nuclei [3].

Disease onset typically occurs during adulthood, between the ages 30 and 50, although cases of juvenile onset do occur. Life expectancy after disease onset is generally 10–20 years [4]. The polyCAG mutation is located in the coding region of the *ATXN1* gene on chromosome 6 [4]. Normal alleles contain 6–44 CAG repeats, along with 1–3 CAT interruptions [5, 6]. Pathogenic alleles require a minimum expansion of 39 repeats [7]. Alleles containing 36–44 CAG repeats interrupted by CATs are typically non-pathogenic, whereas a similar range of CAGs without interruptions generally confer pathogenesis [7, 8]. Age of onset is inversely correlated with disease severity and trinucleotide repeat length. Moreover, disease severity and trinucleotide repeat length often increase in subsequent generations, a form of repeat instability that is indicative of genetic anticipation [4].

The CAG mutation of SCA1 presents a unique case study for polyglutamine repeat disorders, as it is one of only two polyglutamine disorders known to be mediated by interruptions in the repeat tract [9]. This review aims to synthesize the intergenerational and intra-generational manifestations of repeat instability in SCA1, as well as the effect of CAT/histidine interruptions at the DNA and protein level.

## Intergenerational expansions in SCA1: anticipation

### Introduction

A unique phenomenon of trinucleotide repeat disorders is intergenerational instability, often manifesting as anticipation, or the worsening of the disease in subsequent generations. The molecular theory underlying anticipation became clear after the initial identification of trinucleotide repeat expansions in Fragile X Syndrome [10]. A simple explanation seemed to appear: the longer the repeat mutation, the more severe the disease. Consequently, the mechanisms underlying the non-Mendelian inheritance patterns of CAG repeat disorders are of interest. In particular, the size and direction of the instability appears to be influenced by parental sex, focusing research on anticipation towards germline changes in sperm.

### Paternal versus maternal transmission

On the heels of the Fragile X and subsequent Huntington’s discovery (Huntington’s Disease Research Collaborative, 1993) was the observation in SCA1 that there are larger repeat expansions within paternally transmitted ataxin-1 (*ATXN1*) genes. Orr et al. first noted in 1993 that all known juvenile cases of SCA1, like HD, were paternally inherited [4]. Further research by Chung et al. quantified the difference between maternal and paternal transmission of SCA1 in regard to the size of the repeat expansion. They found that 63% of paternal transmissions resulted in an expansion of the CAG repeat sequence, on average by +3.3 repeats. Maternal transmission, on the other hand, resulted in no change or a contraction of the CAG repeat sequence 69 % of the time; on average, maternal transmission resulted in a contraction by −0.4 repeats [11]. Jodice et al. found that expansions larger than 54 repeats were only transmitted by males. On average, paternal transmissions resulted in an increase of +1.75 CAG repeats and maternal transmissions resulted in a contraction of −0.5 CAG repeats [12]. In an investigation of the frequency of meiotic instability in transmission of SCA1, Ranum et al., calculated that roughly 82% of male transmissions and 60 % of female transmissions result in instability [13]. Therefore, the *direction and frequency* of instability differs by sex.

### Male gametic instability

Mosaicism refers to an individual having genetically diverse cells within the same tissue [14, 15]. In respect to SCA1, ATXN1 genes harbor differentially sized repeat expansions. Chong et al. conducted PCR analysis of blood and sperm samples from two SCA1 affected males and demonstrated that SCA1 alleles in sperm cells display greater instability than blood cells [15]. Zulkhe et al. investigated mosaicism in four SCA1 affected males and found mosaicism in the sperm of one; however, the researchers were unable to reach conclusions regarding the other individuals due to degraded samples [16]. Therefore, although based on limited data, there was early molecular evidence for instability of *ATXN1* in the male germline.

Evidence for male gametic instability influencing the expansion of CAG repeats can be found through Huntington’s disease (HD) studies. Telenius et al. [17] investigated paternal transmission of HD, and sought to identify if a relationship exists between the extent of mosaicism and intergenerational CAG repeat expansions [17]. A significant association was found between the level of spermatic mosaicism in the male proband and the direction of intergenerational CAG repeat changes between the parent and the proband; lower levels of mosaicism correlated with CAG repeat contractions and greater mosaicism correlated with CAG repeat expansions or no change [17]. Therefore, the clinical evidence that paternal transmissions result in larger CAG expansions is supported by the molecular evidence of gametic mosaicism in males.

### Developmental timing of expansions

As outlined in Usdin et al.’s [18] review, there are multiple theoretical time points at which the intergenerational repeat expansions could occur: sperm cell precursor division, sperm cell maturation, meiosis, post-meiotic maturation, and early embryonic development [18]. Currently, experimental evidence suggests expansions may be forming at all of these time points.

In their investigation of expansions in transgenic HD mice, Kovtun and McMurray [19] found that the most expansions occurred during the transition from spermatid, an immature haploid cell, to spermatozoa, a mature haploid cell [19]. The investigators concluded that the post-meiotic expansions in mouse sperm cells must be occurring during gap repair of double or single strand breaks. Human HD sperm cells, however, showed expansions both before and after meiosis. The variance in results could be due to the increased number of mitotic divisions that human cells undergo compared to mice cells [18, 20]. Thus, it appears that intergenerational expansions in mice occur during post-meiotic repair pathways, whereas expansions in humans may occur during replication, replicative-repair pathways, or post-meiotic repair pathways.

Embryonic development may also be influencing expansions in transgenic HD mice [21]. Kovtun et al. [21] mated a single transgenic HD male with six non-HD females. The trinucleotide repeat sequence expanded in male offspring and contracted in female offspring. Given that the HD allele originated from the same germ cell line, male and female progeny were expected to display the same distribution of repeat sizes if expansion occurred in sperm cells. Rather, the study findings suggest that early embryonic development plays a role in the formation of intergenerational trinucleotide repeat expansions.

Further research is needed to discern the specific developmental mechanisms that cause expansions and contractions in male and female alleles. Conceivably, a greater understanding of these mechanisms could provide practical insight for genetic counseling and family planning.

## Intragenerational expansions of SCA1: somatic mosaicism

### Introduction

Along with germline instability, somatic instability of ATXN1 is seen in SCA1. As in sperm cells, variations in CAG repeat length appear in other body tissues. The degree of instability in SCA1 varies among tissues in a non-random pattern. Interestingly, this variable instability is found among post-mitotic neuronal tissues, indicating that expansions are arising by non-replicative mechanisms such as transcription or repair. Additionally, the non-random patterns of somatic mosaicism in neuronal tissues highlight a possible connection between neuronal vulnerability, disease, and somatic mosaicism. Age of disease onset may also be associated with somatic mosaicism.

### Neuronal vulnerability

Somatic mosaicism was first characterized in HD, a CAG repeat disease, in 1994 [14]. Telenius et al. quantified the level of somatic mosaicism of the expanded HD allele in five adult late-onset HD patients. Larger expansions correlated with greater mosaicism [14]. Furthermore, the degree of mosaicism ranged from high levels in the basal ganglia and cerebral cortex, intermediate levels in the blood and liver and low levels in the cerebellum. Additionally, greater instability was found in sperm than in blood [14]. The results of this pioneering study provide evidence that somatic mosaicism in HD is organ specific and not solely dependent on the amount of cell division the tissue undergoes. Their findings regarding mosaicism in the brain led to the conclusion that mosaicism parallels neuropathology, as the basal ganglia and cerebral cortex (the brain regions most vulnerable in HD) had the highest levels of mosaicism [14].

Studies analyzing somatic mosaicism in SCA1 soon followed. Chong et al. [15] and Zühlke et al. [16] investigated somatic instability of the expanded SCA1 allele in blood, sperm, and neuronal tissues. Chong et al.’s study found that for two male patients, CAG repeats are more unstable in sperm than in the blood, supporting the HD results [14, 15]. Additionally, investigation of four SCA1 patients demonstrated that the neuronal patterns of mosaicism in SCA1 mirrored those of HD. The greatest instability was seen in the cerebrum and the least was seen in the cerebellum. This finding contradicts the HD hypothesis; namely, tissue mosaicism does *not* correlate with neuronal vulnerability. Zühlke et al. corroborated this result in one brain sample; however, due to degradation of the other samples, they were unable to complete further verification [16]. Subsequent research revealed similar patterns of neuronal mosaicism in HD, SCA1, SCA3, and DRPLA, despite different neuropathologies and neuronal vulnerabilities [22].

To investigate whether the extent of cell division correlates with mosaicism, single-cell analysis was employed. Repeat expansion was quantified in specific cerebellar cells in early- and late-onset DRPLA tissue [23]. The findings reveal that expansions were more common in Purkinje neurons than granule neurons regardless of age of onset. White matter glial cells displayed more expansions than Purkinje neurons in late-onset tissue, but equivalent levels of expansion in early-onset tissue. The study was performed on a single early-onset case and a single late-onset case, so more tissue may be needed to make definitive conclusions. However, the results of the single cell analysis study argue that even within a brain region, the number of mitotic cycles is not a determining factor of somatic mosaicism [23].

More recently the correlation between neuronal vulnerability and somatic mosaicism was investigated in SCA1 “knock-in” mice [24]. The knock-in has 154 CAG repeats (154Q) “knocked” into one copy of the endogenous mouse allele while the unaltered mouse allele houses two CAG repeats (2Q). Expansions up to 56 repeats and contractions greater thvarying extents of mosaicisman 56 repeats were found in the striatum and spinal cord; however, in the cerebellum no expansions larger than 20 repeats were detected [24]. The cerebellum and spinal cord are primarily vulnerable in SCA1, yet the tissues display varying extents of mosaicism indicating a lack of direct relationship between mosaicism and selective neuronal vulnerability in SCA1. Additionally, repeat instability was found to be age-dependent. At 7 weeks, the knock-in allele was stable; at 30 weeks, small expansions and contractions were detectable. And, the majority of larger expansions occurred after 30 weeks following the onset of neuronal dysfunction, further indicating that repeat instability is not directly correlated to neuronal vulnerability [24].

### Age of onset

Somatic mosaicism has also been implicated in modifying age of disease onset in HD. In a correlative analysis of HD patients with extreme early and late onset, cortical instability of the diseased allele was associated with earlier disease onset [25]; the mean maximum expansion for the early onset group (42 repeats) was significantly greater than the mean of the late onset group (29 repeats). No effects on age of onset were found for the normal allele [25].

Despite the findings in HD, somatic mosaicism may not correlate with age of SCA1 onset given the lack of instability seen in the cerebellum. Therefore, tissue-specific factors, rather than disease mechanisms, may cause the cortical and striatal instability found in multiple trinucleotide diseases [25].

## Mechanisms of CAG repeat expansion

### Introduction

As illustrated above, CAG repeat tracts demonstrate both germline and somatic instability. Germline instability has important consequences for inheritance of trinucleotide disorders, as disease severity is correlated to CAG repeat length. The implications of somatic expansions are still unclear; however, neuronal instability may be related to disease onset or disease severity [25, 26]. Because instability in both germline and somatic tissue plays an important role in disease expression, it would be beneficial to identify if expansions are generated by similar mechanisms in all tissue types, or whether germline and somatic instability occur independently. Although the specific pathways by which expansions and contractions are generated are still unclear, an underlying feature of CAG instability, hairpin formations in DNA, has been identified and investigated [27].

### Secondary structure

The genomic DNA secondary structures of trinucleotide repeat sequences were first noted by Gacy et al. in 1995. CAG repeats and their complement CTG strands can bond intra-molecularly to form single-stranded hairpins in vitro. The hairpins that form contain a mismatched base pair every three nucleotides (at the A or T). It is important to note that CTG hairpins are more stable than CAG hairpins due to stronger stacking interactions [27]. A DNA duplex containing a sequence of CAG/CTG repeats can sometimes form hairpins on both strands of the duplex, which is referred to as a “slipped-stranded structure” [28]. A third potential secondary structure is an R-loop, a RNA-DNA hybrid molecule that forms during transcription [18].

The formation of these secondary structures and subsequent expansion of the CAG repeat tract has been hypothesized to occur during a variety of metabolic processes. These intra-strand secondary structures can theoretically form whenever a single strand of DNA is exposed during DNA replication, transcription, repair, or recombination [18, 26]. Disruption of normal processing ensues, often times resulting in a change in size to the CAG repeat sequence.

### Recombination-based mechanisms

In theory, meiotic recombination should provide an opportunity for germline changes to trinucleotide repeat length. However, exchange of homologous flanking sequences have only been observed with CGN repeats [28]. Mitotic recombination via double strand break repair, which does not involve flanking sequence recombination, may play a greater role in trinucleotide repeat instability [18].

### Replication-based mechanism

As reviewed in Mirkin [28], the first proposed model of CAG repeat instability was replication based [29, 30]. Formation of secondary hairpin structures could easily cause misalignment of the DNA strands, which when replicated would result in daughter DNA strands with different sized CAG repeat lengths. If the hairpin forms on the nascent strand, extra repeats could become incorporated into the daughter DNA strand. If the hairpin forms on the template strand, replication machinery could skip past the hairpin, resulting in a contraction of the daughter strand [28, 31]. Importantly, during DNA replication the Okazaki initiation zone on the lagging strand provides a segment of exposed single stranded DNA that could form a hairpin, should it contain a repeat sequence. A hairpin in the Okazaki initiation zone would cause the replication fork to stall and either skip the hairpin resulting in a contraction or reverse and restart, resulting in an expansion [28, 32, 33].

### Repair-based mechanisms

As reviewed in Usdin et al., multiple repair pathways have been shown to be implicated in trinucleotide repeat expansions. The most well characterized pathways are mismatch repair (MMR), base excision repair (BER), and nucleotide excision repair (NER) [18]. Of these, the MMR pathway appears to be most commonly implicated, as there is evidence to suggest cross-talk between the MMR and BER or NER pathways [18]. Additionally, double strand break repair may conceivably play a role in trinucleotide instability via homologous recombination or non-homologous end resection given that both pathways involve the use of DNA synthesis machinery. However, the current evidence regarding the roles of these pathways in producing CAG instability remains inconclusive [18].

Experimental evidence has identified a role for the MMR pathway in both germline and somatic instability. The canonical role of MMR is to repair mismatched base pairs and extra-helical insertion and deletion loops. Hairpins may provide an obstacle to that normal function [34]. Manley et al. [35] investigated the role of the MMR pathway in HD by crossing MutS Homolog 2 (Msh2) deficient mice (*Msh2* ^−^/^−^) with transgenic HD mice. Msh2, in a heterodimer with either Msh6 or Msh3, recognizes mismatches in the DNA, initiating the MMR cascade. Their results show a statistically significant reduction in somatic mosaicism in the liver, cerebellum, cortex, hippocampus, olfactory bulb, striatum, and thalamus of *Msh2* ^*−*^ */* ^*−*^ HD mice compared to HD transgenic mice [35]. Additionally, Msh2 deficiency abrogated age-dependent expansions and contractions [35]. This work supports the notion that the MMR pathway is critical to HD somatic CAG repeat expansion.

Kovtun and McMurray [19] found that the Msh2 is also implicated in germline instability. Following a similar protocol, the researchers mated transgenic HD mice with Msh2 knockout mice and found that in addition to the abrogation of age-dependent somatic expansion in the tail, brain, liver, and spleen, germline expansion was also eliminated [19]. The involvement of Msh2 in both germline and somatic expansions is indicative of the same mechanism creating instability in both types of tissue.

To further investigate the role of the MMR pathway in germline and somatic instability, Wheeler et al. [36] crossed HD Q111 knock-in mice with Msh2 deficient mice. Striatal instability was abolished, confirming earlier results. Maternal germline instability was unaffected. However, the direction of paternal germline instability was affected suggesting that MMR plays tissue-specific roles in CAG repeat expansion. Specifically, Msh2-deficient HD knock-in mice demonstrated contractions in paternal germ cells while HD knock-ins harboring wildtype Msh2 alleles demonstrated expansions and contractions in paternal germ cells [36]. This finding further implicates Msh2 in the expansion of CAG repeat tracts and denotes that an alternative pathway may be responsible for the contraction of those tracts.

Recruitment of MMR proteins Msh2/Msh3 to the site of trinucleotide sequences in DNA may affect the fidelity of repair of the oxoguanine glycosylase (OGG1)-mediated BER pathway [28, 37]. Oxidation of nucleotides by H_2_O_2_ treatment, reactive oxygen species or ionizing radiation can induce 8-oxoguanine lesions which are excised by the BER pathway via OGG1. If the excision occurs within a trinucleotide sequence, hairpin structures can form and become stabilized by binding with MSH2/MSH3. The stabilized bound hairpin structure prevents BER pathway proteins from repairing the hairpin correctly. As a result, expansions occur [28, 37]. Experimental evidence supports this scenario. Base oxidation promoted CAG expansion in human HD fibroblasts and knockout of OGG1 in HD transgenic mice abrogated age-dependent repeat expansions in the tail, brain and liver [37]. Oxidative lesions naturally proliferate with age, thus accounting for the direct correlation between age and instability.

### Transcriptional repair-based mechanisms

Transcription offers a non-mitotic mechanism by which tissue-specific alterations can be made to DNA. During transcription the double helix unravels, providing the opportunity for individual strands to take on secondary structure. Studies of induced transcription through CAG repeat sequences have shown contractions in a human fibrosarcoma cell line [38] and contractions and expansions in bacteria, yeast, fly, and mice models [26]. Transcription-induced repeat instability is thought to be mediated by activation of repair pathways due to secondary structure formation [26].

Both the NER and MMR pathways have been implicated in transcription-induced trinucleotide repeat expansion. There are two NER pathways, transcription coupled NER (TC-NER) and global genome NER (GG-NER). The gene Xeroderma Pigmentosum, Complementation Group A (XPA) is required for TC-NER and GG-NER. Xeroderma Pigmentosum, Complementation Group C (XPC) is required only for GG-NER. Knockdown of XPA in a human cell line resulted in reduced contractions; knockdown of XPC had no effect on CAG repeat instability [38]. Follow up studies knocking down additional genes required for the TC-NER pathway caused reduced CAG contraction [39]. Therefore, there appears to be a significant role for the TC-NER pathway, but not GG-NER, in regulating CAG repeat instability [39].

Once it was determined that the MMR and TC-NER pathways played critical roles in transcription-induced CAG repeat instability, crosstalk between the two mechanisms was further investigated. Experimental evidence from knockdown experiments showed multiple MMR, TC-NER, and transcription genes facilitated CAG contractions, however, simultaneous knockdown of those genes did not produce a synergistic effect [38, 39]. The proposed mechanism for transcription-induced contractions therefore occurs sequentially; separation of DNA strands by RNA polymerase II allows hairpins to form, particularly on the non-template strand. The hairpin recruits MMR pathway proteins, stalling RNA polymerase and initiating the TC-NER pathway to cleave the secondary structure and induce subsequent gap filling. During this synthesis step, due to slippage or misalignment, the size of the CAG repeat sequence may be altered [39].

Other mechanistic possibilities for transcription-induced instability exist [18]. R-loop formations, DNA/RNA hybrids caused by the DNA template strand binding to the RNA transcript inside the transcription bubble, can trigger hairpin formations. Additionally, hairpins may form on the non-template strand, further complicating the repair of the R-loop [18]. R-loops likely result in RNA polymerase II stalling, initiation of the TC-NER pathway and recruitment of BER or MMR pathway proteins [18].

Evidence for the role of NER in SCA1 was obtained through mouse studies [40]. XPA, a NER gene required for both GG-NER and TC-NER, was genetically deleted from SCA1 154Q knock-in mice. XPA ^−^/^−^ SCA1 mice displayed reduced mosaicism compared to haploinsufficient controls (XPA ^+^/^−^ SCA1 mice) in the striatum, hippocampus and cerebral tissues; there was no difference in the germline, kidney, or liver. The data suggests that TC-NER-expansion mechanisms are neuronal tissue-specific in SCA1 mice models and involve XPA protein.

### Trinucleotide repeat instability in neuronal populations

To further elucidate the process of selective neuronal mosaicism, expression of replication and repair genes in different regions of the brain were compared. Replication and MMR genes were upregulated in the cerebellum compared to the striatum of both control and HD patients [41] . However, in HD mice models, no correlation was found between MMR expression and instability in either the cerebellum or striatum; additionally, expression levels were similar for both HD and control mice [41]. Upregulation of replication and MMR genes in the cerebellum may confer protection to the cerebellum against instability in HD [14, 35]; however increased expression does not confer protection against neuronal vulnerability in SCA1. Meta-analysis revealed a biphasic model for the regulation of somatic instability. A basal level of MMR expression is required for expansion; however, increases [41] and decreases [35] from basal expression levels can abrogate somatic instability. Further research is necessary to fully define the role of DNA metabolism and transcription in CAG expansion mechanisms.

### Conclusion

The exact pathway and sequence by which CAG repeat expansions form in germline and somatic tissues has yet to be defined. BER, MMR, and TC-NER pathway proteins contribute to repeat instability in somatic tissue of mice models. Only MMR pathway proteins have been implicated in germline instability. Somatic and germline instability may share a common expansion mechanism, likely among the MMR pathway proteins. Comparing data from HD, SCA1, SCA3, DRPLA, and SBMA, alleles with the largest increases in CAG repeats per generation demonstrated the most somatic mosaicism [22], thereby implicating a common effector.

## Histidine interruptions

### Introduction

Critical to the discussion of trinucleotide repeat disorders is the role of interruptions in the repeat sequence. Interruptions are found in unexpanded alleles for SCA1, SCA2, and Fragile X-Associated Disorders (FXD). In SCA1, CAT trinucleotides, which code for histidine residues, interrupt the polyCAG expansion tract of the *ATXN1* gene. In SCA2, the CAG tract is interrupted by an alternative codon for glutamine, CAA. In FXD, a polyarginine disorder, the normal *FMR1* (also referred to as *FRAXA*) gene contains AGG arginine interruptions throughout its CGG arginine repeat tract [9]. This phenomenon is thought to confer stability to the unexpanded alleles [9]. Interruptions throughout the repeat tract of trinucleotide disorders have been shown to play an important role in mediating hairpin formation and protein aggregation.

### Interruptions in normal alleles

The repeat configuration of normal SCA1 alleles was first characterized in 1993 [11]. Out of 126 normal SCA1 chromosomes analyzed, 123 contained at least one CAT interruption with 18 CAG repeats in the longest continuous tract. No CAT interruptions were found in any of the 30 expanded SCA1 alleles analyzed [11]. This finding provoked the hypothesis that loss of CAT interruptions in expanded SCA1 alleles predisposes the alleles to further expansion [11].

Three potential repeat configurations were identified as normal SCA1 alleles [11]. The configuration (CAG)_n_CATCAGCAT(CAG)_n_ accounted for 78% of normal alleles, (CAG)_n_CAT (CAG)_n_ accounted for 11% and (CAG)_n_CATCAGCATCAGCAT(CAG)_n_ accounted for 4%. Further study confirmed similar repeat configuration distributions in a Polish population and suggested that pathogenic alleles may generate from a lengthening of the 5′ tract followed by a loss of the CAT interruption [42].

Analysis of intermediate SCA1 alleles (36–41 repeats) pinpointed the threshold pathogenic length to 39 repeats [7]. Interruptions were always present when the tract contained fewer than 39 CAGs and always absent when the tract contained 41 or more CAGs. Of the five identified alleles containing 39 repeats, one housed a CAT interruption. Disease phenotype only manifested in the individuals containing pure repeat tracts [7]. Moreover, CAT interruptions modulated SCA1 pathogenesis at the threshold level, highlighting the clinical relevance of identifying CAT interruptions during diagnosis [7].

### Interruptions in expanded alleles: effect on age of onset

Other novel interruption configurations have been identified, including expanded alleles with CAT interruptions. An expanded tract with the sequence (CAG)_12_CATCAGCAT(CAG)_12_CATCAGCAT(CAG)_14/15_ was identified in two separate case studies. Of the four patients harboring this expanded allele, three were asymptomatic, including a 66-year old male, which is past the usual age of disease onset. The fourth patient presented with neurological symptoms at age 2, but juvenile SCA1 was ruled out. In both case studies, the interrupted allele was transmitted stably through a paternal lineage [6, 43]. These findings suggest that CAT interruptions in expanded alleles can delay age of onset, or even eliminate onset altogether. Moreover, these case studies raise the possibility that the longest uninterrupted tract may be a better indicator for disease onset, given that a repeat tract of 12 CAGs would not correspond to presentation of SCA1 symptoms [6].

Further supporting this hypothesis, a third case study identified an SCA1 patient with the repeat sequence of (CAG)_45_CATCAGCAT(CAG)_10_ who began experiencing symptoms at age 50 [44]. The authors statistically determined that an expanded allele with 58 CAG repeats should correspond to disease onset at 22, whereas 45 repeats should correspond to a disease onset at 41.8 years. Therefore, it appears that the longest uninterrupted CAG repeat tract is a better predictor for age of onset [44].

In a cohort of 35 SCA1 patients, four individuals possessed expanded alleles with CAT interruptions [8]. There was a greater correlation with age of onset and the length of the longest uninterrupted CAG repeat stretch (R^2^ = 67%) than with the full repeat tract length (R^2^ = 21.2%). Therefore, CAT interruptions in expanded alleles, which appear with an allele frequency of 6–11%, appear to have a significant protective effect [8, 44]. Furthermore, an improved correlation was found between length and aggregation state of the polyQ peptide when the longest uninterrupted CAG repeat tract was applied in the analysis [8]. Consequently, SCA1 diagnosis would benefit from the inclusion of CAT trinucleotide identification when sequencing alleles [8].

### CAT interruptions: DNA and RNA level effects

A potential mechanism by which CAT interruptions may promote stability is through regulation of DNA secondary structure. Slipped-strand structures, S-DNA, can form from trinucleotide repeat DNA sequences following denaturation and re-duplexing [9]. Re-duplexing reactions were performed on six different DNA clones, four uninterrupted (30, 49, 60, and 74 CAGs) and two interrupted, (30 and 44 CAGs), to encourage S-DNA formation. Among the uninterrupted clones, the percentage and structural complexity of S-DNA increased with CAG length. Comparison of the expanded clones with equal CAG length (44 CAG uninterrupted vs 44 CAG interrupted) revealed decreased S-DNA in the interrupted clone, while comparison of the unexpanded clones (30 CAG uninterrupted vs 30 CAG interrupted) yielded no significant difference in S-DNA percentage [9]. Despite minor variations in repeat length and CAT trinucleotide number, the results of the study strongly suggest that CAT interruptions could impact the tendency towards S-DNA formation.

Meta-analysis of clinical data confirmed that alleles below the pathogenic threshold containing CAT interruptions were stably transmitted. Larger alleles without interruptions were transmitted stably and unstably [9]. Additional case studies provided evidence of stable transmission of pure expanded CAG repeat tracts, through maternal and paternal lineages [7, 45], as well as unstable maternal transmission of an interrupted pathogenic SCA1 allele, resulting in the contraction of the allele to a pure repeat tract [8]. Therefore, although CAT interruptions generally confer stability to CAG repeat tracts, exceptions do exist.

There are at least three possible mechanisms by which interruptions may limit strand slippage, thus promoting genetic stability. First, interruptions may make inter-strand slippage unfavorable, given that mismatched base pairings would result [9]. Second, interruptions specifically in FMR1 (FRAXA) alleles have demonstrated reduced hairpin stability, thus intra-strand interactions may be inhibited by interruptions [9, 27]. Last, interruptions may reduce slippage by limiting the slip-out structures that can form; perhaps hairpin formations are confined to short pure repeat tracts or to conformations where CAT interruptions flank the hairpin [9].

In a detailed investigation of RNA transcript stability, Sobczak and Krzyzosiak [46] characterized the structural differences between interrupted and uninterrupted SCA1 transcripts. Pure repeat tracts of pathogenic lengths were shown to form a singular hairpin with a clamped flanking sequence, providing additional stabilization to the hairpin. Non-pathogenic sequences containing interruptions took on different conformations depending on the location of the interruptions within the sequence. Symmetrically located interruptions were incorporated into the terminal loop of the hairpin structure. Asymmetric interruptions either induced the formation of smaller internal loops or branched hairpin structures. Moreover, when asymmetrically interrupted pathogenic transcripts adopted these alternative structures, the length of the pure CAG repeat hairpin stem fell below the pathogenic threshold. Shortening of the hairpin structure to below the pathogenic threshold in some interrupted transcripts offers a possible explanation for reduced penetrance of interrupted expanded SCA1 alleles [46].

### Histidine interruptions–protein level effects

The hallmark pathology of SCA1 are nuclear inclusions of aggregated ataxin-1 protein in some, but not all, vulnerable brain regions [47]. The findings that histidine interruptions can mediate age of onset and disease severity [6, 43] warrant further investigation on the effect of interruptions on ataxin-1 protein conformation and aggregation.

#### Beta-sheet structure

In an in vitro comparison of secondary structure conformation, aggregates of expanded and unexpanded versions of both interrupted and pure polyQ peptides adopted *β*-sheet conformation [48, 49]. However, the observed circular dichroism spectra indicated that for both the short and long interrupted peptides, the *β*-sheets were intra-molecularly hydrogen bonded with the histidine residues found at the head of the hairpin. In contrast, the uninterrupted peptides adopted tightly linked, wide intermolecular *β*-sheets [48, 49]. Additional alkylation experiments determined that the histidine residues in the interrupted peptide aggregates are solvent-accessible, fitting with the previously proposed structural model [48, 49]. However, it remains possible that the histidine residues could be incorporated into the *β*-sheet itself, with its side chains simply projecting outwards [50].

Additional structural analysis included investigation of monomeric interrupted and uninterrupted peptides. In their monomeric forms, uninterrupted and interrupted peptides of pathogenic length display unordered/flexible random coil conformations [8]. Treatment of aggregates to induce disaggregation resulted in unordered structure regardless of the peptide [49]. Taken together, the results suggest that histidine residues may alter the aggregation kinetics of polyQ structures at the polymer level, without imparting additional order at the monomer level.

#### Aggregation kinetics

Multiple techniques have been used to analyze the aggregation properties of polyQ peptides. The first aggregation experiments comparing interrupted and uninterrupted polyQ peptides measured solubility and light scattering [48, 49]. Interrupted constructs, both at pathogenic and non-pathogenic lengths, demonstrated reduced aggregation potential compared to their uninterrupted counterparts. Histidine interrupted Q_22_ peptides displayed greater solubility than pure polyQ peptides of the same length [48]. Maximum solubility occurred when only one glutamine resided in between the histidine residues (HQH) as compared to four glutamines in (HQ_4_H) [48]. At pathogenic lengths, time-dependent Rayleigh scattering indicated that interrupted Q_42_ peptides present less scattering than pure Q_42_ peptides, indicating a lower aggregation potential of interrupted peptides [49].

In a recent, detailed study, ten different peptide configurations were compared including interrupted and pure Q_30_, Q_54_, and Q_82_ peptides along with four different interrupted configurations of the length Q_64_–Q_69_. Histidine interruptions were found to reduce peptide aggregation in transfected COS cells. Moreover, the Q_64_ configuration containing six histidine residues reduced aggregation more substantially than the other interrupted configurations of the same length containing fewer histidine residues [8]. Not only do the presence of histidine residues mitigate aggregation, but the quantity of histidines impacts aggregation propensity. Further evidence was provided by filter retardation studies. Pure and interrupted polyQ tracts of similar lengths (pure Q_47_ and interrupted Q_45_) demonstrated comparable levels of protein aggregation, both aggregating more than an interrupted Q_32_ construct [51]. Uninterrupted Q_45_ and Q_82_ peptides aggregated to a greater degree than interrupted peptides of the same lengths, indicating that interruptions do significantly affect aggregate formation [8]. The minor discrepancy in results between the two studies may be due to the location of the histidine within the repeat tract. However, taken together, both length of the tract and the presence and quantity of histidines regulate the aggregation dynamics of polyQ peptides.

In agreement with the findings suggesting reduced aggregation stability of interrupted constructs, histidine interruptions were found to promote a reduction of aggregation rates [50]. Thioflavin-T (ThT)-fluorescence and solubility assays demonstrated that interrupted Q_30_ peptides aggregate at a slower rate than uninterrupted peptides of the same length. Analysis of nucleation kinetics led to the conclusion that the mechanism of aggregation is identical for interrupted and uninterrupted polyQ peptides at neutral pH [50]. However, thermodynamic stability of the kinetic nucleus was greatly reduced with the insertion of histidines. The calculated 20-fold reduction in the nucleation equilibrium constant and a slight reduction in the elongation equilibrium constant provide a potential mechanism for the reduced aggregation rate of histidine-interrupted, non-expanded polyQ peptides [50].

This difference in aggregation rate is also detected in pathogenic polyQ peptides. ThT-fluorescence and light scattering assays of interrupted and uninterrupted Q_41_ peptides demonstrated reduced aggregation for the interrupted peptide [8]. Additionally, monitoring of light scattering as a function of temperature determined that the histidine-interrupted aggregates yield a melting point 3°C lower than uninterrupted aggregates. This temperature difference suggests that the slower aggregation rate of histidine-interrupted constructs is a result of reduced stability [8].

Additionally, fibril formation and seeding effects of interrupted and uninterrupted peptides have been investigated. Ribbon/plate-like fibril structures are seen for uninterrupted and interrupted polyQ peptides, both above and below the pathogenic threshold length [50, 52]. However, in addition to plate-like structures, at neutral pH, interrupted aggregates also form long filaments [50]. Both peptides were equally efficient at seeding interrupted and uninterrupted peptides; therefore, monomeric recruitment appears to be unaffected by the presence of histidines [8, 50]. Collectively, histidine interruptions may induce differences in fibrillar morphology that pose a kinetic barrier to aggregation, rather than differences in monomer recruitment rates.

## Conclusions

Trinucleotide repeat instability in SCA1 manifests as both inter- and intra-generational instability, demonstrating high levels of mosaicism in the male germline, striatum, and spinal cord. CAT interruptions of the CAG repeat tract provide a stabilizing effect, mediating secondary structure formation, the cornerstone of trinucleotide repeat expansion mechanisms. DNA repair pathways, namely MMR, have been implicated in both germline and somatic instability, although the exact process still has yet to be defined.

Further investigation of expansion mechanisms in somatic tissues and germline is needed to identify whether all repeat instability is induced by the same mechanism. Intergenerational instability poses a threat to increasing disease severity in SCA1; therefore, identifying the mechanism of expansion has great clinical significance. In vitro findings have identified a role for the MMR, BER, and NER pathways in facilitating trinucleotide repeat instability. Currently, the only in vivo evidence from mice models for repair based expansion mechanisms suggest a role for the NER pathway in SCA1, and the MMR and BER pathways in HD. Future research should aim to clarify whether all three pathways are implicated in SCA1. However, it is important to keep in mind that the in vivo mouse work suggesting functional mechanisms for specific repair proteins have been conducted in isolated and controlled situations (i.e. animal over-expression and knockout models). The application to human disease pathology remains unclear. Importantly, mice do not naturally develop neurodegenerative disease due to aggregation-resistant proteins homologous to human aggregation-prone counterparts [53] and due to the relatively short lifespan of mouse neurons. The biological response seen in experimental mice as a result of an induced phenotype may operate by different mechanisms than the response in humans.

In regard to somatic instability, much of the known research was obtained from HD studies. Initial findings suggest that eliminating somatic instability via Msh2 deficiency results in delayed nuclear accumulation of Huntingtin protein, the characteristic hallmark of HD pathology [36]. Additionally, somatic instability was found to correlate with HD disease onset [25]. However, given the unique role of histidine interruptions in conferring stability in SCA1, comparisons between SCA1 and other CAG repeat disorders must be made with caution. The role of somatic instability in modifying disease onset and severity in SCA1 specifically still needs to be defined.

Furthermore, trinucleotide disorders can be organized into two categories- those that contain repeat tracts in the coding region and those that contain repeats in the non-coding regions. In this review, CAT(U)/histidine interruptions have been shown to significantly impact DNA and RNA secondary structure formation and protein aggregation. The unique structural effects created by interruptions suggests that we must begin to consider a third subset of trinucleotide disorders when investigating trinucleotide expansion mechanisms, those that contain interruptions.

The topic of nuclear inclusions remains a lingering question in the SCA1 field. The lack of ATXN1 inclusions in cerebellar Purkinje nuclei despite Purkinje neuronal vulnerability calls into question the role of protein accumulation in disease pathogenesis [54]. Unique to the cerebellum, and when compared to striatum and cortex, the CAG repeat tract of SCA1 alleles is relatively stable in humans [15, 16] and mice models [24]. Prevention against neuronal instability may be due to upregulation of DNA repair and replication genes in the cerebellum [41]. The cerebellum, despite its substantial vulnerability to neurodegenerative disease, maintains repeat stability and precludes the formation of nuclear inclusions in Purkinje neurons. Perhaps the absence of nuclear inclusions and somatic stability in the cerebellum are linked, due to the upregulation of DNA repair and replication genes and/or other cerebellum-specific factors?

## Abbreviations

ATXN1: Ataxin-1
BER: Base excision repair
GG-NER: Global genome NER
HD: Huntington’s disease
MMR: Mismatch repair
Msh2: MutS Homolog 2
NER: Nucleotide excision repair
OGG1: Oxoguanine glycosylase
SCA1: Spinocerebellar ataxia type 1
TC-NER: Transcription coupled NER
